# Supplementary material for: Pathogenic FANCC Variants Are Associated with Accessory Breasts in a Sub-Saharan African Multiplex Family
Source: Curr Issues Mol Biol. 2025 Oct 22;47(11):875. doi: 10.3390/cimb47110875 (PMC12650935; doi:10.3390/cimb47110875)
Supplement: Supplementary file 1 [file cimb-47-00875-s001.zip › cimb-3939390-supplementary.pdf]

SUPPLEMENTARY MATERIALS

SUPPLEMENTARY FIGURES

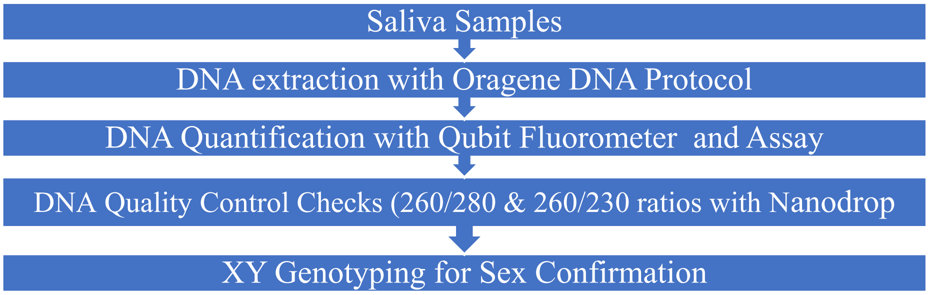

Figure S1: DNA processing workflow prior to sequencing.

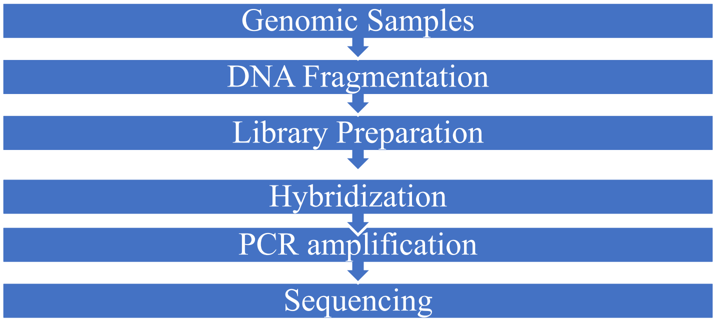

Figure S2: A detailed workflow of the whole exome sequencing.

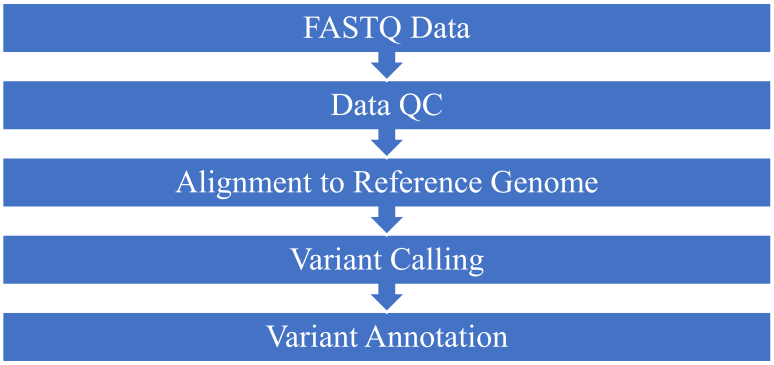

Figure S3: The variant calling workflow depicting various processes involved in generating the VCF files.

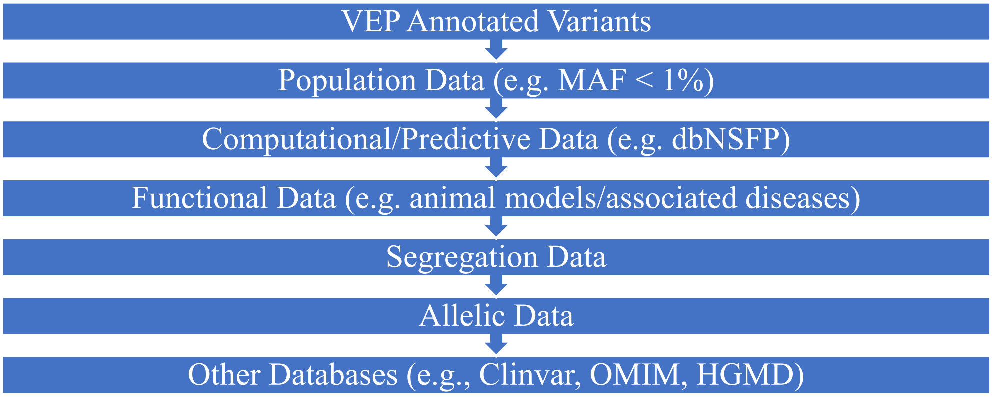

Figure S4: A detailed workflow of the variant prioritization process.

SUPPLEMENTARY TABLES

Table S1: Predictive threshold of variant effect prediction tools.

Tool	Range	Predictive threshold
ClinPred	0–1	>0.5
MetaRNN	0–1	>0.5
BayesDel_addAF	-1.11707–0.750927	>0.0692655
REVEL	0–1	>0.5
CADD	0–50+	>20
AlphaMissense	0–1	Pathogenic (>=0.564-1), Ambiguous (0.34-0.56), Benign (<0.34)
MutPred	0–1	>0.5
PolyPhen2	0–1	>0.5
MutationAssessor	-5.17–6.49	High (>3.5), Medium (1.935-3.5). >=1.935 (to cater for both medium and high
MutationTaster_rankscore	0–1	>0.31733
SIFT	0–1	<0.05

Table S2: Sample sequencing statistics of the multiplex family.

Sample ID	# Reads	Yield (Mbases)	Mean Quality Score	%Bases>=30
GH20222001-1	42,408,589	12,723	35.89	93.51
GH20222001-2	57,848,189	17,354	35.89	93.54
GH20222001-4	29,561,957	8,869	35.92	93.71
GH20222001-5	51,182,010	15,355	35.80	93.02
GH20222001-6	39,423,422	11,827	35.76	92.78
GH20222001-7	49,652,098	14,896	35.84	93.25

Table S3: Summary statistics of alignment.

Sample ID	Total cleaned reads	Unique reads	% Unique reads	% Unique reads aligned	% Target regions >=20X
GH20222001-1	83,563,528	57,014,482	68.23	99.89	97.50
GH20222001-2	114,024,312	79,208,491	69.47	99.67	97.76
GH20222001-4	58,316,832	41,845,591	71.76	99.69	97.13
GH20222001-5	100,707,930	69,336,772	68.85	99.88	97.67
GH20222001-6	77,524,506	55,272,435	71.30	99.64	97.56
GH20222001-7	97,808,792	66,690,615	68.18	99.67	97.67

Table S4: Variant effect Predictors for assessing effects of variants.

Genename (rs_dbSNP)	Chromosome position	Ensembl Transcript id	HGVSc VEP	HGVSp VEP	SIFT score	Polyphen2 score	Mutation Taster score	Mutation Assessors score	MetaRNN score	REVEL score	MutPred score	BaysDel_addAF score	ClinPred score	ClinVar	CADD score	Alpha Missense score
PRSS50 (rs145256818)	Chr3: 46714301	ENST00000460241	c.671C>T	p.Pro224Leu	0.042	0.999	0.50806	2.72	0.577448	0.677	NA	-0.043982	0.244417	NA	24.7	0.5082
FANCC (rs7669094)	Chr9:95172132	ENST00000289081	c.360del	p.His120GlnfsTer24	NA	NA	NA	NA	NA	NA	NA	NA	NA	NA	24.6	NA
FANCC (rs750003253)	Chr9:95172134	ENST00000289081	c.355_358del	p.Ser119IlefsTer24	NA	NA	NA	NA	NA	NA	NA	NA	NA	NA	27.3	NA
SLC7A7 (rs764284986)	Chr14: 22813019	ENST00000397532	c.380T>C	p.Ile127Thr	0.001	0.988	0.81001	2.22	0.806096	0.783	NA	0.148079	0.700941	Uncertain significance	24.6	NA
NDE1 (Novel)	Chr16: 15687426	ENST00000396355	c.438C>G	p.Ile146Met	0.003	1	0.58761	3.205	0.813465	0.576	0.498	0.363148	0.984266	NA	14.12	0.6037
DIP2B (rs147225936)	Chr12: 50731373	ENST00000301180	c.3646T>C	p.Tyr1216His	0	0.704	0.81001	2.485	0.753665	0.733	NA	0.034214	0.602101	Uncertain significance	26.1	0.5254

ADGRG6 (rs1204304460)	Chr6: 142415804	ENST00000367609	c.2678G>A	p.Arg893Gln	0.069	1	0.81001	NA	0.797845	0.427	NA	0.143504	0.99134	NA	33	0.3949
CHDH (rs765283936)	Chr3: 53823630	ENST00000315251	c.379G>A	p.Val127Ile	0.016	1	0.81001	2.59	0.15804	0.303	0.743	-0.252456	0.217853	NA	27.7	0.2117
ACKR2 (rs577087357)	Chr3:42865498	ENST00000422265	c.1010_1016del	p.Pro337LeufsTer21	NA	NA	NA	NA	NA	NA	NA	NA	NA	NA	23.1	NA
C3orf62 (rs868287641)	Chr3:49276455	ENST00000343010	c.416_417del	p.Glu139GlyfsTer20	NA	NA	NA	NA	NA	NA	NA	NA	NA	NA	24.3	NA

OR4Q3 (novel)	Chr14:19747835	ENST00000642117	c.436_449del	p.Asn146ProfsTer32	NA	NA	NA	NA	NA	NA	NA	NA	NA	NA	26.9	NA
OR2W1 (rs149813138)	Chr6: 29044316	ENST00000377175	c.860C>T	p.Pro287Leu	0.013	1	0.81001	2.4	0.023775	0.623	NA	-0.207743	0.095184	NA	26.5	0.3402
MYO1H (rs200225794)	Chr12:109447180	ENST00000310903	c.3115T>C	p.Ter1039ArgextTer1	NA	NA	NA	NA	NA	NA	NA	NA	NA	NA	20.3	NA

RYR1 (rs61739911)	Chr19:38512366	ENST00000359596	c.9355C>T	p.Arg3119Cys	0	0.014	0.4977	2.41	0.084122	0.603	NA	0.112095	0.153397	Conflicting_classifications_of_pathogenicity	25.7	0.5758
TTR (rs1555631393)	Chr18:31595139	ENST00000237014	c.220G>A	p.Glu74Lys	0.016	0.994	0.81001	3.325	0.947781	0.86	0.794	0.497176	0.980634	Pathogenic	28.3	0.6044

All transcripts are canonical transcripts. N/A: not applicable. Red: Deleterious; Green: tolerable; NA: Not available.
